# An assessment of data quality and sociodemographic variation in health service utilisation of general practice, emergency department and admitted services in a New South Wales linked health data asset: a retrospective cohort study of Lumos

**DOI:** 10.1136/bmjopen-2025-102055

**Published:** 2025-07-22

**Authors:** Tristan Bouckley, Rimma Myton-Katieva, David Peiris, Devaki Nambiar, Samuel Prince, Simon Bishop, Damien Cordery, Flynn Robert Hill, Patricia Correll, Anne-Marie Feyer, Gill Schierhout, Anna Campain

**Affiliations:** 1 Faculty of Medicine and Health, The George Institute for Global Health, University of New South Wales, Sydney, New South Wales, Australia; 2 Faculty of Medicine and Health, University of New South Wales, Sydney, New South Wales, Australia; 3 Faculty of Medicine and Health, The George Institute for Global Health, New Delhi, India; 4 Prasanna School of Public Health, Manipal Academy of Higher Education, Manipal, India; 5 System Information and Analytics Branch, New South Wales Ministry of Health, Sydney, New South Wales, Australia

**Keywords:** Health policy, Health Services, Electronic Health Records

## Abstract

**Abstract:**

**Objectives:**

This study aimed to (1) assess Lumos data quality, a New South Wales (NSW) statewide linked health data asset; and (2) determine sociodemographic variation in health service utilisation of general practice, emergency department and admitted services.

**Design:**

A retrospective cohort study using Lumos, a linked health data asset.

**Setting:**

A representative statewide sample population of NSW, Australia.

**Participants:**

People residing within NSW with an electronic health record at a Lumos participating general practice between January 2010 and June 2023.

**Primary and secondary outcome measures:**

Data quality indicators of Lumos including completeness, representativeness against NSW population data, consistency and timeliness. Furthermore, variation in general practice visits, emergency department presentations and hospital admission rates stratified by age, sex, rurality and Index of Relative Socio-economic Disadvantage (IRSD)—a measure of socioeconomic status used in Australia, where lower values represent greater relative disadvantage across a range of metrics such as education and income.

**Results:**

At the time of analysis, Lumos included records from 5.2 million unique patients, representing half (49.7%) of the NSW resident population. Limiting data to 2022, the Lumos population distribution broadly aligned with the 2021 Census except for IRSD quintile four and five which were under-represented (15.0% vs 20.4% (standardised difference −0.14)), and over-represented (29.7% vs 19.9% (standardised difference 0.23)), respectively. Age and greater relative disadvantage were associated with higher rates of general practice visits and hospital admissions. Greater relative disadvantage was also associated with higher rates of emergency department presentations.

**Conclusions:**

Lumos’s ability to overcome historical limitations of separately managed health data in Australia and its demonstrated data quality present an opportunity to enhance health system policy and planning in NSW. The variation in service utilisation across primary and tertiary care by population and geography apparent in Lumos reinforces the need for tailored service planning.

**STRENGTHS AND LIMITATIONS OF THIS STUDY**

- Analysis draws on comprehensive linked primary and tertiary health records from over five million unique patients, overcoming historical division of health data in Australia.
- The approach allows for the simultaneous comparison of health service use, including general practice, emergency department and hospital admissions with a consistent population.
- Provides unique and comprehensive insights on health system utilisation across the state of New South Wales, Australia, stratified by key sociodemographic variables including age, sex, remoteness and Index of Relative Socio-economic Disadvantage—a measure of socioeconomic status.
- Potential gaps in Lumos participating general practices (practices that do not supply patient records for linkage to Lumos) may lead to pockets of poor data quality.
- Other measures exist that could further contribute to validation of Lumos data quality, particularly when considering specific health conditions and other demographics.

## Background

 Health system management relies on high-quality data to support operational functions, performance and predict future service demand.^1 2^ Administrative health data and electronic medical records provide the backbone for health system management,^3^ supporting planning, funding and workforce recruitment and training. Appropriate disaggregation of these data allows for a closer understanding of health burdens, health seeking and outcomes thereof in populations that may face unique or even shared disadvantages.^4^

The Australian health system is operated under a complex mixed model arrangement composed of both public and private healthcare providers overseen by federal, state and territory and in certain areas local government.^5^,^7^ Public healthcare is mostly governed and managed by the state government, which operates and regulates secondary and tertiary health services, while the federal government funds private-run primary and specialist care under a universal health insurance scheme (Medicare), limiting comprehensive whole-of-health system oversight.^8 9^ For this reason, there has historically been limited capability to determine health system utilisation across the two systems.

Established in 2019, the New South Wales Ministry of Health (NSW Health) in partnership with NSW Primary Health Networks (PHNs) developed Lumos. Lumos is an enduring health data asset that functions as a health information exchange,^10 11^ linking NSW Health held administrative health data with primary care electronic health record (EHR) data.^12^ Over the past 3 years, Lumos has rapidly expanded general practice (GP) integration to present a comprehensive data asset that overcomes many of the historical challenges that previously prevented the integration of GP and hospital health data. This presents significant opportunities to strengthen understanding of health system requirements and service demand in Australia’s most populous state.

To date, there have been limited linkages of population-wide primary, secondary and tertiary healthcare data in Australia. Where this has occurred, these have often been limited to specific disease conditions and populations. The Lumos data asset presents an opportunity to provide a particularly critical infrastructure to support the health system in managing more complex long-term conditions arising from an ageing population, where at least 27% of the population experience a chronic condition requiring complex healthcare support.^13 14^ At the time of writing, other major population-wide linked datasets are emerging across Australia, such as the National Health Data Hub, Person Level Integrated Data Asset, PeopleWA and SA NT DataLink.^15^,^18^ Each is shaped in a unique way and connected to different collections of data. Lumos is a NSW-specific initiative that uniquely links GP data with other parts of the health system. As these datasets expand and others are made available across Australia, understanding the data quality within each and unique contributions each data asset provides will become increasingly important to ensure health policies and service design are appropriately informed.

The quality of the data used to inform health system management and performance is critical to ensuring appropriate decision-making.^19^ No dataset is perfect and biases risk impacting interpretation.^20^ For these reasons, regular, systematic assessments of data quality and the underlying data structures are important to ensure a shared understanding of the limitations and generalisability of the data.^21^ Data quality assessment is particularly important for routinely collected data that are primarily captured to support the provision of patient care, rather than the secondary purpose of health system management and evaluation.^1 20 21^

Due to the ongoing expansion of the Lumos data asset, and the need to better understand how populations engage with the health system across state and federally funded health services, our study aimed to (1) assess the data quality of Lumos for the NSW population and (2) determine sociodemographic variation in health service utilisation of GP, emergency department and admitted services.

## Methods

### Design and setting

A retrospective cohort study was undertaken of all NSW residents with an EHR at a Lumos participating GP.

### Data sources

Lumos comprises patient records across several datasets linked to the index GPEHR dataset. Linked datasets include admitted patient data collection (APDC), emergency department data collection (EDDC), NSW non-admitted patient data collection(NAP), NSW mental health ambulatory care data collection (MH-AMB), NSW Ambulance data collection and NSW Registry of Births, Deaths and Marriages death registrations among others.

For each Lumos extract, deidentified patient-level records are linked using Privacy Preserving Record Linkage so no patient identifying information is captured. Correll *et al*^12^ previously reported on the structure of Lumos and data linkage processes, which has since incorporated additional administrative health datasets and expanded patient records. In this study, we used the seventh Lumos data extract, which included health records up to 30 June 2023, derived from 628 GPs across NSW. Publicly available data on numbers and distribution of GPs in NSW were provided by HealthDirect,^22^ a national virtual public health information service. We also drew from government documents and publications, including data dictionaries and Lumos protocols to capture data structure information.

### Data quality measure selection

To date, there is ongoing discussion on appraisal of electronic health data quality, with various frameworks, terms and approaches considered.^120 21 23^,^26^ Despite inconsistencies in terminology and data quality assessment approaches, there is growing alignment on key overarching domains that we have adopted for this study—these include (1) completeness, (2) representativeness (external concordance), (3) consistency and (4) timeliness.^1 20 23^ Online supplemental appendix 1 details definitions used in this study for each domain and the measures used.

Within each domain, different measures of data quality are used depending on the task at hand.^26^ For this reason, while retaining the overarching core quality assessment domains, we have tailored data quality measures relevant to sociodemographics (age, sex, Index of Relative Socio-economic Disadvantage (IRSD), rurality and other geographical measures including local government area (LGA)) and service utilisation variables for the purpose of health system planning and monitoring. IRSD is a measure of socioeconomic status used in Australia, whereby lower values represent greater relative disadvantage derived from a range of metrics such as education and income.^27^ We included both patient and patient encounter level data to provide a comprehensive assessment of data completeness and appropriateness.^26^

### Statistical analysis

#### Data quality

To determine the overall NSW population captured in Lumos, we calculated the proportion of the total Lumos population against the estimated population residing in NSW between 2011 and 2022. The estimated resident population was calculated using the NSW population from 2011 and adding annual births and domestic and international migration to NSW through to 2022 from reference data.^28^

We calculated the population distribution of Lumos active patients in 2022 and compared the reported prevalence with the NSW population estimates reported in the Australian Census 2021. Lumos active patients were defined as patients who had at least one record within GP EHR, APDC, EDDC, NAP, MH-AMB or NSW ambulance datasets in 2022. We then calculated the standardised difference to determine the magnitude of difference between Lumos and Census^29^—an approach used to overcome sensitivity that other tests experience when using large data.^30^ We used an absolute standardised difference of 10% as an indicator of an imbalance.^29^

We stratified our analyses by sex, age (0–17, 18–54, 55–74, 75 years and above), rurality, IRSD quintiles (quintile 1 represents people residing within the most disadvantaged areas, through to quintile 5, which represents people residing within the least disadvantaged areas) and LGA. We used a standard measure of rurality, allocating the population to major cities, inner regional, outer regional and remote.^31^

We also used visual inspection measures to detect outliers and uniformity, including an age-sex population pyramid and geospatial mapping. Lastly, we mapped GP distribution by LGA to contextualise our results.

#### Service utilisation

Using 2022 data, we calculated service utilisation summary statistics for hospital admissions, ED presentations and GP attendance, stratified by age, sex, rurality and IRSD. Service utilisation for each service was defined as the number of records each person had for each service type. We calculated rates per 100 person-years using Lumos active patients within 2022. Patients who died during 2022 contributed a half-year, assuming equal distribution of deaths across the year.

We modelled health service utilisation rates using multiple negative binomial regression analyses adjusting for age, sex and IRSD. We generated adjusted rate ratios (ARRs) with 99% CIs for GP doctor or nurse visits, hospital admissions and ED presentations. Regression analysis was performed on complete cases. Data manipulation and analyses were conducted using Microsoft SQL Server Management Studio V.18^32^ and R V.4.3.2.^33^ Figures were produced in R V.4.3.2, using R packages.^34 35^

The NSW Centre for Health Record Linkage (CHeReL) undertakes linkage in accordance with NSW and Commonwealth ethical, legal, privacy and confidentiality requirements.^36^

#### Patient and public involvement

Patients and/or the public were not involved in the design, or conduct, or reporting, or dissemination plans of this research.

## Results

### Data completeness

The Lumos data asset contained 5.2 million unique patients between 2010 and 2023, compared with an estimated 10.4 million unique individuals residing in NSW between 2011 and 2022.^28^ Thus, at extraction, the Lumos dataset contained 49.7% of the NSW resident population since 2010. There were 3.3 million active individuals in Lumos in 2022 (table 1), equating to 41% of the estimated NSW population in 2021. Population counts by service type across the history of Lumos are provided in online supplemental appendix 2. Across all active patient entries in 2022, we found at most 0.3% missing data on demographics for sex, rurality, IRSD or age (table 1).

**Table 1:** Standardised difference between Lumos active population 2022 and Census 2021

Population characteristics	Lumos population N (%)	2021 Census N (%)	Standardised difference*
All	3 294 991 (100)	8 072 163 (100)	–
Sex			
*Male*	1 529 079 (46.4)	3 984 166 (49.4)	−0.06
*Female*	1 761 797 (53.4)	4 087 995 (50.6)	0.06
*Missing*	3902 (0.1)	–	–
Rurality categories			
*Major cities*	2 568 801 (78.0)	6 080 428 (75.3)	0.06
*Inner regional*	544 423 (16.5)	1 569 737 (19.5)	−0.08
*Outer regional*	149 568 (4.5)	377 693 (4.7)	−0.01
*Remote (including very remote)*	21 711 (0.7)	32 927 (0.4)	0.03
*Missing*	10 488 (0.3)	–	–
IRSD quintiles			
*1 (most disadvantaged)*	599 066 (18.2)	1 589 014 (19.8)	−0.04
*2*	581 283 (17.6)	1 589 467 (19.8)	−0.06
*3*	630 742 (19.1)	1 618 049 (20.2)	−0.03
*4*	495 136 (15.0)	1 635 157 (20.4)	*−0.14*
*5 (least disadvantaged)*	977 752 (29.7)	1 598 619 (19.9)	*0.23*
*Missing*	11 012 (0.3)	–	–
Age groups (years)			
*0–17*	655 351 (19.9)	1 749 217 (21.7)	−0.04
*18–54*	1 607 909 (48.9)	3 937 014 (48.8)	0.00
*54–74*	712 919 (21.6)	1 750 515 (21.7)	0.00
*75 and older*	318 206 (9.7)	635 402 (7.9)	0.06
*Missing*	100 (0.0)	–	–

* A difference of 0.1 or above is considered a meaningful difference and is highlighted in italics.

### Representativeness (external concordance)

The sex, rurality and age distribution of the active Lumos population was broadly representative of the 2021 NSW population reported in Census^37^ table 2(table 1). However, we were unable to confirm representativeness of the two least disadvantaged IRSD quintiles. Lumos captured less people in quintile four than the 2021 Census (15.0% vs 20.4% (standardised difference −0.14)), and captured more people in the highest quintile (least disadvantaged) compared with Census 2021 (29.7% vs 19.9% (standardised difference 0.23)).

**Table 2:** ARRs of utilisation by subpopulations for each service type

Total records	General practice attendance		All hospital admissions		ED presentations	
	N (%)	ARR (99% CI)	N (%)	ARR (99% CI)	N (%)	ARR (99% CI)
All	17 398 698 (100)		1 257 389 (100)		1 573 287 (100)	
Sex						
Male	7 442 511 (42.8)	–	593 522 (47.2)	–	760 723 (48.4)	–
Female	9 947 035 (57.2)	1.17 (1.16 to 1.17)	663 680 (52.8)	1.02 (1.01 to 1.03)	812 284 (51.6)	0.93 (0.92 to 0.93)
IRSD (1—most relatively disadvantaged areas; 5—least relatively disadvantaged areas)						
1	3 436 714 (19.8)	1.12 (1.12 to 1.13)	261 838 (20.8)	1.31 (1.30 to 1.33)	370 002 (24.5)	1.94 (1.93 to 1.96)
2	3 187 554 (18.3)	1.03 (1.03 to 1.04)	241 265 (19.2)	1.19 (1.18 to 1.20)	347 388 (22.1)	1.88 (1.86 to 1.89)
3	3 319 245 (19.1)	1.04 (1.03 to 1.04)	246 277 (19.6)	1.18 (1.16 to 1.19)	325 691 (20.7)	1.62 (1.61 to 1.64)
4	2 516 600 (14.5)	1.01 (1.00 to 1.01)	179 573 (14.3)	1.08 (1.06 to 1.09)	212 164 (13.5)	1.35 (1.33 to 1.36)
5	4 905 398 (28.2)	–	324 339 (25.8)	–	312 880 (19.9)	–
Age groups (years)						
0–17	2 506 830 (14.4)	0.90 (0.89 to 0.90)	112 340 (8.9)	0.64 (0.63 to 0.65)	339 615 (21.6)	1.15 (1.14 to 1.16)
18–54	6 983 609 (40.1)	–	430 936 (34.3)	–	723 587 (46.0)	–
54–74	4 677 089 (26.9)	1.53 (1.52 to 1.54)	386 376 (30.7)	2.06 (2.04 to 2.08)	288 121 (18.3)	0.89 (0.89 to 0.90)
75 and older	3 229 759 (18.6)	2.44 (2.43 to 2.45)	327 737 (26.1)	4.21 (4.16 to 4.26)	221 964 (14.1)	1.64 (1.62 to 1.66)

Each service type was modelled separately, and adjusted for age, sex and IRSD.

General practice attendance was limited to doctor or nurse encounter.

ARR, adjusted rate ratio; ED, emergency department; IRSD, Index of Relative Socio-economic Disadvantage.

**Figure 1:**
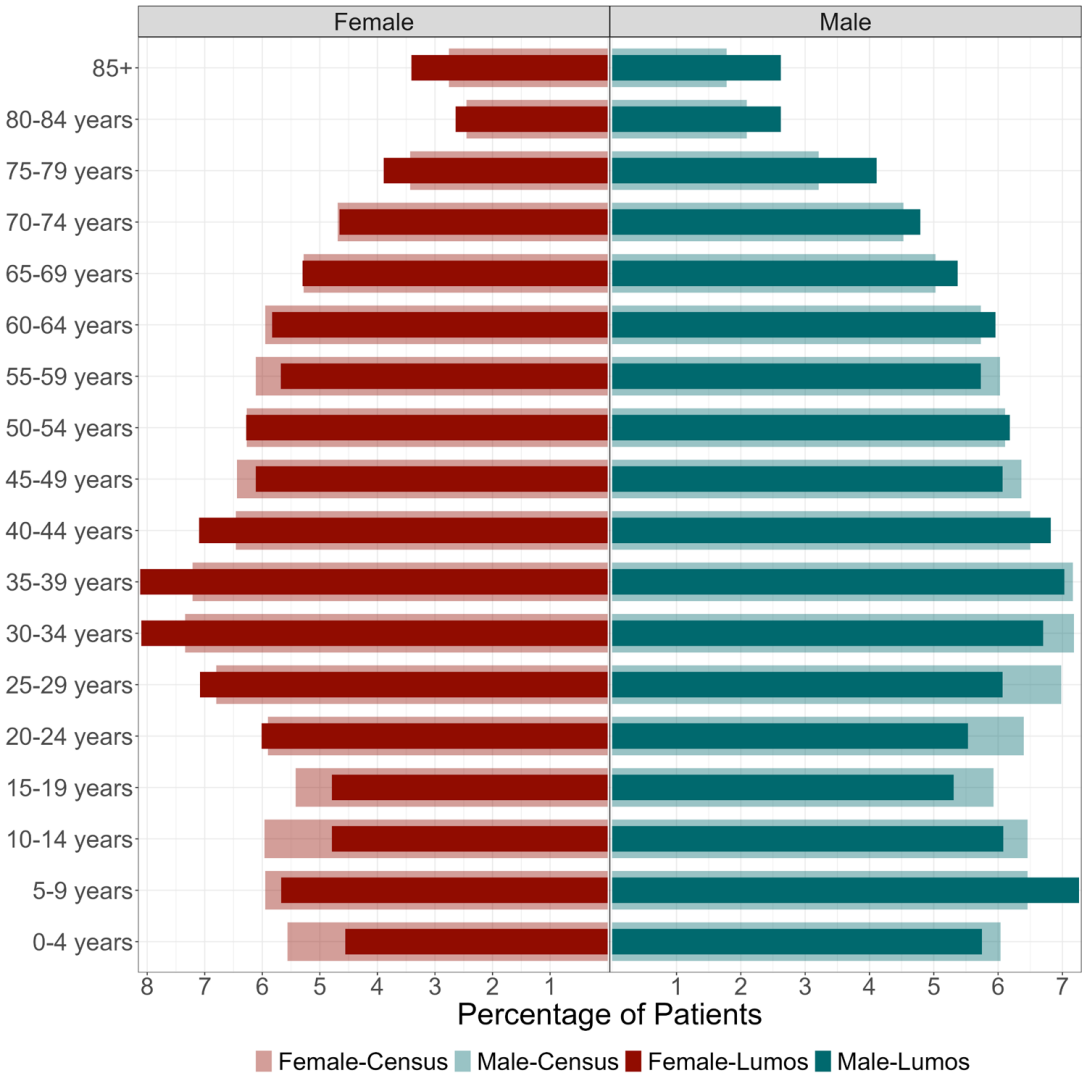
Age and sex distribution of active Lumos population 2022 and Census 2021.^37^

The population pyramid demonstrated good representation for most age brackets (figure 1). However, there appeared to be a slight over-representation of females aged 30–44 and over 85, and males aged 5–9 and 75 and older. Slight under-representation is apparent for males aged 15–34 and females aged 10–19.

**Figure 2:**
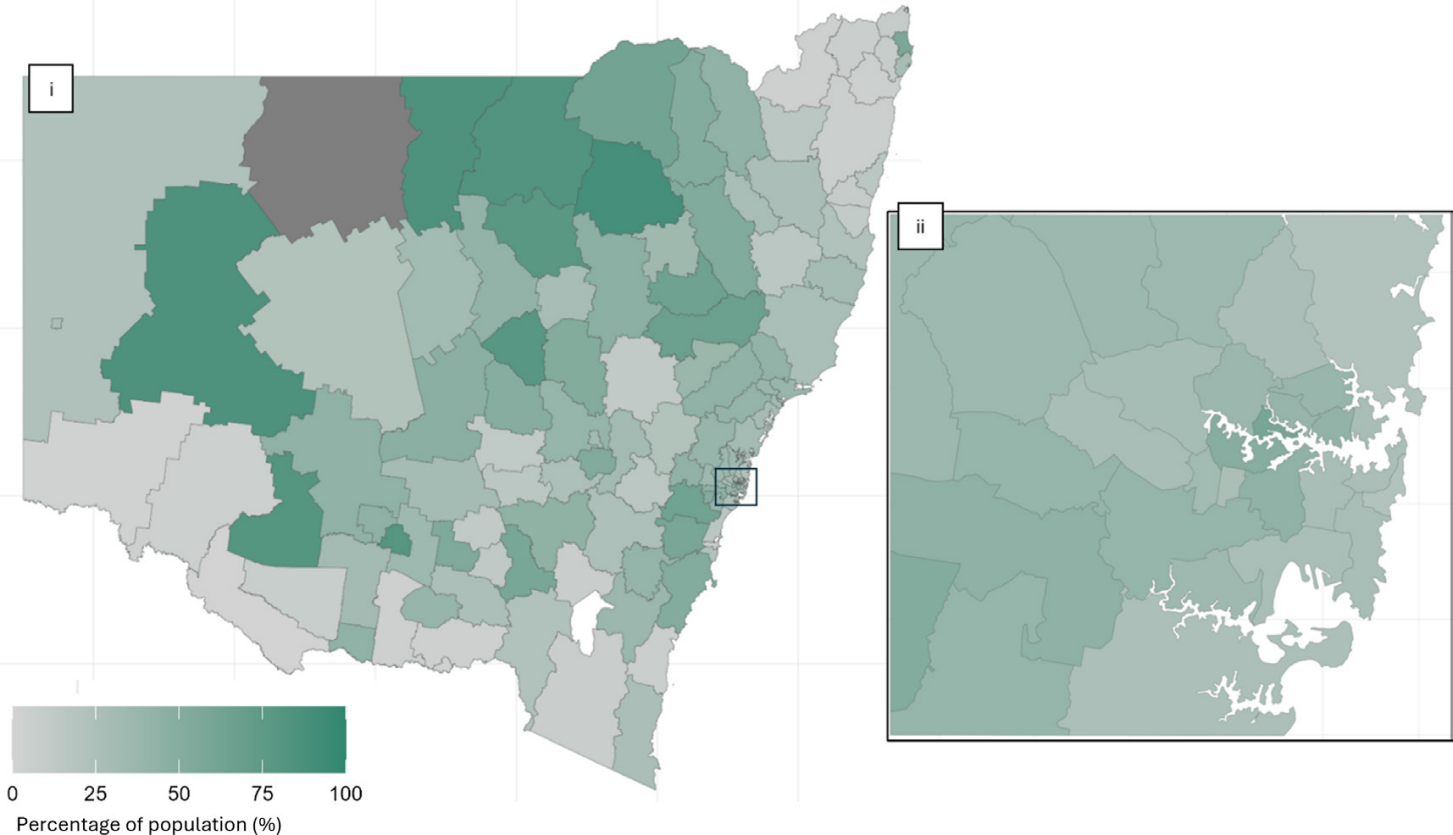
Proportion of NSW population included in Lumos in 2021 by local government area population estimates. (**i**) NSW. (ii) Greater Sydney region. NSW, New South Wales.

Greater Sydney had a relatively stable proportional distribution of the estimated LGA population (figure 2). However, regional and remote areas had greater variability, with particularly low capture in Southwest and Northeast NSW (in light grey). Other regional areas captured close to 100% of the estimated population (deep green in colour). Bourke (presented in dark grey) was an exception to the analysis and reported above census population estimates.

Few LGAs showed significant under-representation or over-representation compared with Census 2021 (see online supplemental appendix 3), with only three LGAs with a standardised difference greater than 0.1.

Lumos enrolled GPs accounted for 26.6% of publicly reported GPs,^22^ and over 70% of LGAs across NSW have at least one GP enrolled in Lumos (map included at online supplemental appendix 4). Major city areas tended to have a greater consistency of Lumos enrolled GPs, while outer regional and remote areas often had either high or low coverage.

### Consistency

Age, sex, IRSD, and rurality were captured in Lumos with definitions standardised and consolidated at the person-level within a summary table to overcome differences in duplicated variables captured between datasets (online supplemental appendix 5). The Lumos data dictionary has a complete definition for each variable except for sex, which varied across each dataset (summary at online supplemental appendix 6). To overcome inconsistencies across the datasets that make up the Lumos data asset, sex was defined by the patient’s most recent reported sex and prioritised by dataset to preference stronger datasets such as APDC, where it is known to more accurately and consistently capture demographic information. As a result of this process and the variable underlying dataset definitions used, trans and gender diverse populations are not reported and only 213 people were coded as indeterminate in any of the datasets. For all considered measures, there were no unexplained variables in the Lumos dataset.

The age variable reported in Lumos differs from Census 2021 where date of birth is missing. In the Census, missing age values are imputed and calculated using an age distribution of the population, while Lumos draws from multiple datasets to minimise missing data. Finally, the approach to defining IRSD and rurality aligns with Census 2021, with the exception for GP EHR. GP EHR determines location based on postcode, while the other datasets used a more granular geographic representation—statistical area Level 1 (SA1).

We also examined consistency between datasets within Lumos and found that patient service entries in the Lumos data asset were consistent across each dataset we measured except for the MH-AMB dataset. This is due to gaps in recording attendance at large group services in MH-AMB.

### Timeliness

**Figure 3:**
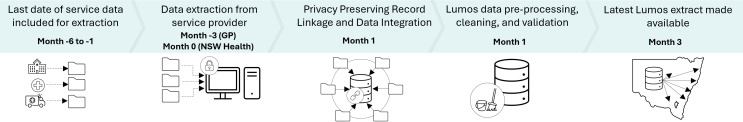
Timeliness of Lumos availability.

Due to the multistep process to establish the Lumos data asset, there is a lag time between the last reported patient episode of care in a Lumos dataset and data availability for analysis (figure 3). We found that the minimum lag time was between four and ten months for the larger datasets such as APDC, EDDC, MH-AMB and GP EHR, while some smaller datasets had greater delays.

### Health service utilisation

The Lumos active population in 2022 accounted for close to 17.4 million GP presentations, 1.3 million hospital admissions and 1.6 million ED presentations (online supplemental appendix 7). Across all service types, females made up a greater proportion of service users and this proportional difference was particularly pronounced for GP attendance (57.2% vs 42.8%). Furthermore, people aged 75 and above accounted for 26.1% of hospital admissions, despite only accounting for 9.7% of the Lumos active population.

Among the active Lumos patients in 2022, there were 530 GP presentations, 38 hospital admissions and 48 ED presentations per 100 person-years, respectively (online supplemental appendix 8). When adjusting for age and sex across all services, people living in more relatively disadvantaged areas had progressively greater GP attendance rates, hospital admissions and ED presentations (table 2). GP presentation and hospital admission rates progressively increased with age. Females had 17% higher GP attendance rates than males (ARR 1.17, 99% CI: 1.16 to 1.17), yet 7% lower rates of ED presentations (ARR 0.93, 99% CI: 0.92 to 0.93).

## Discussion

We demonstrated that Lumos provided broadly strong data quality across key population metrics and measures. Importantly, Lumos is largely representative of the NSW population for age, sex and remoteness. IRSD was representative with the exception of quintiles 4 and 5 (populations residing within the least disadvantaged areas). This does not limit its utility as adjustments can be made to compensate for this variation. We also demonstrated the growing opportunities to understand patient and population-wide service utilisation, bridging the historical divide between state and federal health data for health policy, planning and evaluation.

We demonstrated variation in service utilisation patterns and geographic location. Concurring with other studies,^38 39^ age was the single largest predictor of service utilisation across GP and hospital admissions. Overall, hospital admission rates in Lumos aligned with NSW statewide hospital admission rates.^40^ The alignment between our findings on hospital admissions with other reports provided another measure of external concordance. Like others,^41^ we found lower GP attendance rates in more remote areas; however, this was not as large as anticipated. This may be better explained by IRSD, given the moderate correlation between remoteness and IRSD, which would also explain the observed increased ED utilisation among the most relatively disadvantaged populations. Limited access to GP and socioeconomic disadvantage has been reported as drivers of ED utilisation in other studies.^38^

Similar to Correll *et al*,^12^ ED presentation rates were higher in Lumos (48 per 100 population) compared with other NSW reported rates at 36.8 per 100 population.^42^ As Lumos only included people who attended a Lumos enrolled GP, this has the potential to over-report the more health conscious and active health seeking population.^43 44^ This was indicated through a greater representation of females of childbearing age, and likely people with a chronic health condition who will have a greater presence within health services. The Lumos dataset also skews to a slightly older population, which, as we and others have reported, has a higher health service demand.^38 39^ This is in line with findings from the previous Lumos analysis.^12^ While measures have been taken to reduce false-positive links and linkage failures where a true link exists, these continue to be a risk^44 45^ and can also distort average age and sex.^46^ However, the linkage process used by CHeReL uses the same privacy preserving record linkage method demonstrated elsewhere in Australia, which has previously reported alignment of 99.3% of records with other linkage methods.^47^

Consideration needs to be taken when extrapolating Lumos data to certain population groups and regions. Rural areas had greater variability in representation, likely driven by the small number of GPs^48^ and population size, increasing sensitivity to changes. Regions without Lumos enrolled GPs were often located near borders with other states—Queensland and Victoria. Several nearby major border cities, including Mildura and the Gold Coast, are outside of NSW; or in the case of Albury-Wodonga, health is managed by the Victorian government and PHN. The large border cities likely explain low GP enrolment and low Lumos patient representation. A post-hoc analysis of statewide GP data found only 160 (6.8%) of NSW GPs were situated in areas without a Lumos enrolled GP.^22^ The over-representation within the Bourke region may be due to a small but highly mobile population in the region, given the lack of major cities nearby.

As of February 2025, 807 GPs were participating in Lumos, which is over five times as many as in the previous Lumos analysis that was limited to 156 practices.^12^ As a result, the population within Lumos has increased from under 1.5 million (16% of the NSW resident population) to 5.2 million (almost 50% of the NSW resident population). Broadly, and where it is possible to compare, the Lumos dataset has retained similar representativeness across age, sex, IRSD and geography over time,^12^ despite its considerable expansion. This is with the exception of a slight over-representation of people residing in the least disadvantaged areas as discussed earlier. The analysed data extract (extract seven) relied on a standardised approach to demographic characteristic definitions (online supplemental appendix 5). This was initially produced in Lumos to ensure consistency across datasets; nevertheless, it could impact on the visibility of some populations such as trans and gender diverse populations. We note that this has been removed in future iterations of Lumos. However, the poor capture of trans and gender diverse populations within the underlying datasets poses a challenge to appropriate policy development for these populations and continues to foster their invisibility.^49^ There continue to be very real gaps in health system data, with invisibility of various historically marginalised populations perpetuated, as demonstrated by trans and gender diverse population data.^50^ Further investigation is required for subpopulations such as culturally and linguistically diverse populations, among others, as there is substantial variation in service demand within the NSW population that cannot be explained by sex, age, IRSD and remoteness. Where these data are missing, policy will continue to risk perpetuating inequities.^50 51^

### Strengths and limitations

Our study provides one of the largest overviews of health service utilisation by demographic groups across primary and tertiary care in Australia to date, while also demonstrating a measure of trustworthiness of the data through purposeful selection of data quality measures. However, we recognise other measures exist that could further contribute to validation of Lumos data quality, particularly when considering specific health conditions and other demographics. The lack of consensus in measures of data quality continues to challenge standardisation.^1 20 21 52^

While standardised difference is a useful tool to overcome sensitivity issues relating to large data, it may be too insensitive for small numbers at the extreme ends. This may explain why several low-populated LGAs appeared to be significantly under-represented in some rural areas, but did not appear under-represented using standardised difference. We also recognise that Lumos reflects populations accessing GP services within NSW and may not be representative of the broader Australian population.

## Conclusions

Lumos overcomes historical limitations of separately managed health service data between state and federal jurisdictions in Australia, presenting an opportunity to support health system policy and planning. Broadly, age, sex, IRSD and geographic distributions were proportionately captured with minimal missing data, enabling a fit-for-purpose dataset. However, we continue to advise caution for more rural areas, high quintile IRSD populations, and recognition that Lumos reflects populations accessing GP services. The significant variation in service utilisation across primary and tertiary care by population and geography highlights the need for tailored service planning to manage population requirements.

## Supplementary material

10.1136/bmjopen-2025-102055 online supplemental file 1

## Data Availability

Data may be obtained from a third party and are not publicly available.
