# Supplementary material for: An assessment of data quality and sociodemographic variation in health service utilisation of general practice, emergency department and admitted services in a New South Wales linked health data asset: a retrospective cohort study of Lumos
Source: BMJ Open. 2025 Jul 22;15(7):e102055. doi: 10.1136/bmjopen-2025-102055 (PMC12306357; doi:10.1136/bmjopen-2025-102055)
Supplement: online supplemental file 1 [file bmjopen-15-7-s001.docx]

## Appendix 1. Domains of data quality and related measures

| Domain | Definition | Data type | Measure | Operationalisation of measure |
| --- | --- | --- | --- | --- |
| Completeness | Measurement of missing relevant data and whether data available is appropriate for requirements [1]. | Patient | NSW resident population in Lumos [2] | Percent of NSW resident population in Lumos |
|  |  | Patient encounter | Patients within each dataset [2] | Percent of patients within each Lumos dataset (e.g. APDC, EDDC) |
|  |  | Patient | Missing data for demographics (sex, rurality, IRSD, age) [1, 2] | Percent missing data by demographic variable in Lumos, where variable is either blank or recorded as missing. |
|  |  | Patient encounter | Availability of relevant dimensions [3] | Summary statistics on demographics (sex, rurality, IRSD, age) and service type (APDC, EDDC) |
| Representativeness (External concordance) | Alignment or comparability of data and distributions with external reports [3-5] | Patient | Distributional alignment of age and sex with census data [3-6] | Construct age and sex distribution pyramid comparing Lumos with Census 2021. |
|  |  | Patient | Distributional alignment of demographics (Rurality, LGA, IRSD, age and sex) with census data [3-6] | Calculate proportion of Lumos population for each demographic.   - Statistical test: Standardised difference between Lumos and Census 2021 data. |
|  |  | Patient encounter | Agreement of service utilisation with external reports [5] | Calculate annual service utilisation counts and distribution by service (APPDC, EDDC etc) and demographics.   - Statistical test: Multivariate negative binomial regression.   Compare adjusted risk ratios with other published literature and reports. |
| Consistency (conformance) | Data element alignment with standard formatting and definitions [2, 6] | Patient | Demographic data values conform with external standards [2, 6] | Lumos demographic derived variable definition alignment with Census 2021 variable definitions. |
|  |  | Patient | Data values conform to allowable values or ranges [2, 6] | Data value alignment between Lumos and the Lumos data dictionary. |
|  |  | Patient encounter | Data is consistently documented for all encounters [1]* | Consistency of patient service entries reported for each Lumos datasets. |
| Timeliness (currency) | Data is up to date for required tasks [2, 3] | Patient encounter | Speed at which data is available in Lumos [2] | Last available patient entry data for each dataset in Lumos. |
|  |  |  |  | Detail Lumos data extraction and access timeline. |

*Entry could also be reported under completeness, as the measure is related to both completeness and consistency domains.

## Appendix 2. Total unique populations captured within each service

|  | General practice | All hospitals | Public hospitals | Private hospitals | Emergency department | MH-AMB | NAP | NSW ambulance records |
| --- | --- | --- | --- | --- | --- | --- | --- | --- |
| Data availability | Jan 2010 -Jun 2023 | Jan 2010 - Mar 2023 | Jan 2010 - Mar 2023 | Jan 2010 - Mar 2023 | Jan 2010 - Mar 2023 | Jan2010 – Dec 2022 | Jan 2010 - Mar 2023 | Jan 2010 -  Mar 2023 |
| Lumos |  |  |  |  |  |  |  |  |
| *Number of linked patients* | 4,992,180 | 3,135,912 | 2,435,491 | 1,617,946 | 3,199,434 | 345,956 | 3,108,290 | 1,142,114 |
| *Per cent of linked patients* | 96.03 | 60.32 | 46.85 | 31.12 | 61.55 | 6.65 | 59.79 | 21.97 |
| Sex |  |  |  |  |  |  |  |  |
| *Male* |  |  |  |  |  |  |  |  |
| *Number of linked patients* | 2,368,872 | 1,406,364 | 1,095,379 | 704,437 | 1,548,868 | 155,267 | 1,412,352 | 544,664 |
| *Per cent of linked patients* | 45.57 | 27.05 | 21.07 | 13.55 | 29.79 | 2.99 | 27.17 | 10.48 |
| *Female* |  |  |  |  |  |  |  |  |
| *Number of linked patients* | 2,610,516 | 1,729,340 | 1,339,955 | 913,402 | 1,650,343 | 190,564 | 1,695,723 | 597,349 |
| *Per cent of linked patients* | 50.22 | 33.27 | 25.78 | 17.57 | 31.75 | 3.67 | 32.62 | 11.49 |
| Rurality |  |  |  |  |  |  |  |  |
| Major city |  |  |  |  |  |  |  |  |
| *Number of linked patients* | 3,959,857 | 2,432,532 | 1,839,582 | 1,307,902 | 2,414,026 | 246,273 | 2,403,142 | 852,477 |
| *Per cent of linked patients* | 76.17 | 46.79 | 35.39 | 25.16 | 46.44 | 4.74 | 46.23 | 16.40 |
| Inner regional |  |  |  |  |  |  |  |  |
| *Number of linked patients* | 764,490 | 523,275 | 435,588 | 244,612 | 577,123 | 71,395 | 517,433 | 212,357 |
| *Per cent of linked patients* | 14.71 | 10.07 | 8.38 | 4.71 | 11.10 | 1.37 | 9.95 | 4.09 |
| Outer Regional |  |  |  |  |  |  |  |  |
| *Number of linked patients* | 213,256 | 150,269 | 134,340 | 54,617 | 174,446 | 22,932 | 155,857 | 64,384 |
| *Per cent of linked patients* | 4.10 | 2.89 | 2.58 | 1.05 | 3.36 | 0.44 | 3.00 | 1.24 |
| Remote and very remote |  |  |  |  |  |  |  |  |
| *Number of linked patients* | 30,389 | 19,904 | 18,301 | 5,655 | 23,369 | 4,146 | 22,169 | 9,171 |
| *Per cent of linked patients* | 0.58 | 0.38 | 0.35 | 0.11 | 0.45 | 0.08 | 0.43 | 0.18 |
| Age |  |  |  |  |  |  |  |  |
| 0-17 years of age |  |  |  |  |  |  |  |  |
| *Number of linked patients* | 953,934 | 665,594 | 560,856 | 207,302 | 651,251 | 27,626 | 669,120 | 130,238 |
| *Per cent of linked patients* | 18.35 | 12.80 | 10.79 | 3.99 | 12.53 | 0.53 | 12.87 | 2.51 |
| 18-54 years of age |  |  |  |  |  |  |  |  |
| *Number of linked patients* | 2,614,100 | 1,373,337 | 1,049,252 | 697,062 | 1,541,634 | 233,431 | 1,478,254 | 493,766 |
| *Per cent of linked patients* | 50.29 | 26.42 | 20.18 | 13.41 | 29.66 | 4.49 | 28.44 | 9.50 |
| 55-74 years of age |  |  |  |  |  |  |  |  |
| *Number of linked patients* | 985,353 | 703,416 | 497,054 | 452,899 | 649,008 | 58,642 | 620,748 | 261,292 |
| *Per cent of linked patients* | 18.95 | 13.53 | 9.56 | 8.71 | 12.48 | 1.13 | 11.94 | 5.03 |
| 75 years of age and above |  |  |  |  |  |  |  |  |
| *Number of linked patients* | 436,701 | 393,563 | 328,327 | 260,682 | 357,536 | 26,255 | 340,164 | 256,815 |
| *Per cent of linked patients* | 8.40 | 7.57 | 6.32 | 5.01 | 6.88 | 0.51 | 6.54 | 4.94 |
| IRSD (1 – most relatively disadvantaged areas; 5 – least relatively disadvantaged areas) | | | | | | |  |  |
| 1 |  |  |  |  |  |  |  |  |
| *Number of linked patients* | 887,179 | 580,635 | 515,589 | 205,121 | 621,420 | 77,610 | 580,053 | 249,405 |
| *Per cent of linked patients* | 17.07 | 11.17 | 9.92 | 3.95 | 11.95 | 1.49 | 11.16 | 4.80 |
| 2 |  |  |  |  |  |  |  |  |
| *Number of linked patients* | 850,082 | 559,018 | 469,664 | 247,823 | 603,969 | 73,058 | 559,752 | 226,310 |
| *Per cent of linked patients* | 16.35 | 10.75 | 9.03 | 4.77 | 11.62 | 1.41 | 10.77 | 4.35 |
| 3 |  |  |  |  |  |  |  |  |
| *Number of linked patients* | 951,345 | 593,389 | 477,994 | 290,450 | 621,031 | 71,986 | 602,047 | 222,023 |
| *Per cent of linked patients* | 18.30 | 11.41 | 9.19 | 5.59 | 11.95 | 1.38 | 11.58 | 4.27 |
| 4 |  |  |  |  |  |  |  |  |
| *Number of linked patients* | 760,814 | 464,939 | 352,846 | 252,206 | 479,131 | 47,309 | 468,262 | 161,303 |
| *Per cent of linked patients* | 14.64 | 8.94 | 6.79 | 4.85 | 9.22 | 0.91 | 9.01 | 3.10 |
| 5 |  |  |  |  |  |  |  |  |
| *Number of linked patients* | 1,517,123 | 927,603 | 611,422 | 616,983 | 862,874 | 74,717 | 887,995 | 279,212 |
| *Per cent of linked patients* | 29.18 | 17.84 | 11.76 | 11.87 | 16.60 | 1.44 | 17.08 | 5.37 |

## Appendix 3. Standardised difference* in LGA population distribution between Lumos 2022 and NSW 2021 Census


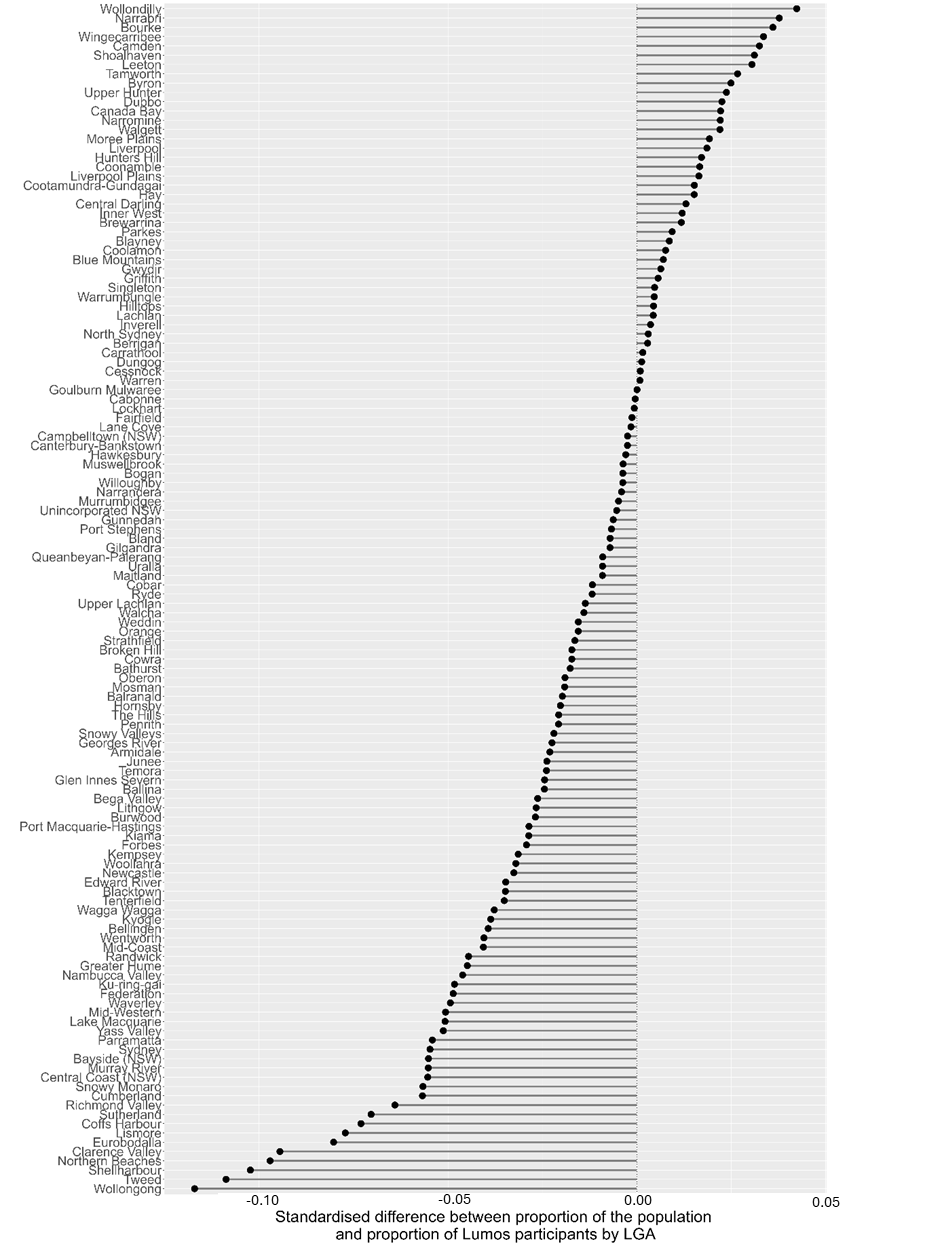


*A difference of 0.1 or above is considered a meaningful difference.

Albury LGA was excluded from the analysis as health system governance is under the Victorian jurisdiction.

Appendix 4. Proportion of Lumos enrolled general practices in each NSW local government area

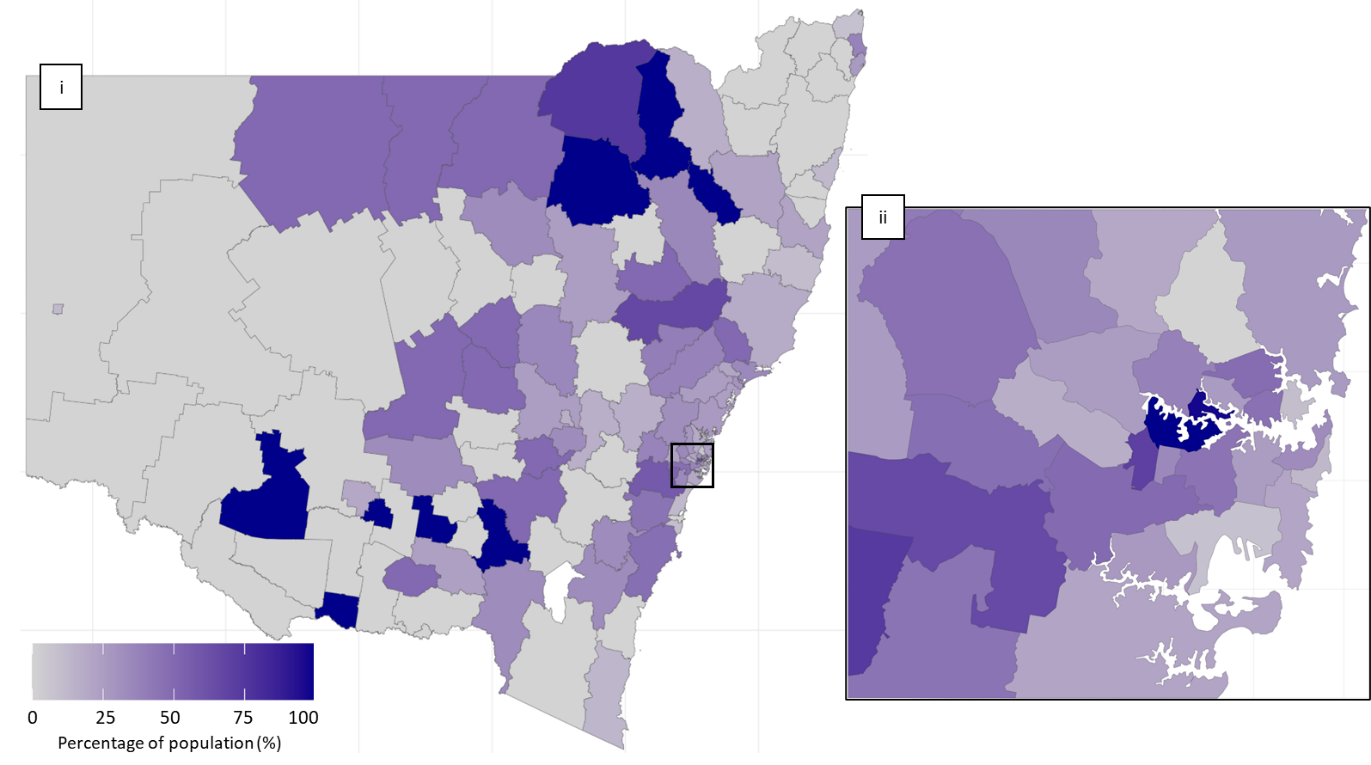


*i. New South Wales; ii. Greater Sydney region*

## Appendix 5. Standardised definitions of patient demographic characteristics consolidated within the Lumos person-level summary table.

| Variable | Stage | Datasets/Source | Definition/Rules |
| --- | --- | --- | --- |
| Sex | 1 | GPEHR, APDC, EDDC, NAP, ICD, Ambulance, MHAMB, CCR | - Derive the most recent known sex recorded in dataset (based on Date of last visit in GPEHR data, Date of diagnosis for NSW Central Cancer Registry (CCR), Entry date for ICD in NSW Integrated Care Database (ICD), and service event date for other datasets). Where duplicates still exist, list sex as 9 – Unknown. |
|  | 2 | Across all datasets | - Following the priority of datasets to consolidate final value of sex - If the variable exists in APDC and is not listed as BLANK/Unknown, take the value recorded in APDC, followed by EDDC > GPEHR > MHAMB > NAP > CCR > ICD > Ambulance. |
| Age | 1 | GPEHR | - If a person has more than 1 record in GPEHR dataset, maximum and minimum year of birth are derived. - If the difference between their maximum and minimum year of birth are more than 5 years and both records have more than 1 encounter/general practice visit recorded, all records related to that PPN (unique identifier for each patient) are flagged to remove. - Else if the difference between their maximum and minimum year of birth are less than 5 years, reference year of birth is extracted from record with the most general practice encounters. |
|  | 2 | ICD | - - In the ICD dataset, the recorded year of birth from the earliest IC enrolment date is retained. - If the difference between the ICD age and NSWPS year of birth are more than 5 years, all records related to that PPN are flagged to remove. - Else if the difference between their ICD and NSWPS year of birth are less than 5 years, reference year of birth is extracted from record with the earliest IC enrolment date. |
|  | 3 | APDC, EDDC, NAP, Ambulance and MHAMB | - Maximum and minimum recorded year of birth of each PPN are derived in each data set. - If the difference between their maximum and minimum year of birth are more than 5 years and they all have more than 1 service event recorded, all records related to that PPN are flagged to remove. - Else if the difference between their maximum and minimum year of birth are less than 5 years, reference year of birth is extracted from the latest service event/contact. - Else if the difference between maximum and minimum year of birth are more than 5 years and one of them only got recorded in 1 service event, reference year of birth is reset to the maximum/minimum derived year of birth with more than 1 service event. |
|  | 4 | CCR | - Maximum and minimum recorded year of birth of each PPN are derived in each data set. - If the difference between their maximum and minimum year of birth are more than 5 years, all records related to that PPN are flagged to remove. - Else if the difference between their maximum and minimum year of birth are less than 5 years, reference year of birth is extracted from the record with latest year of diagnosis. |
|  | 5 | Across all datasets | - Maximum and minimum recorded reference year of birth of each PPN are derived from all sources. - If the difference between their maximum and minimum year of birth are more than 5 years, PPN is flagged to remove. - Else if the difference between their maximum and minimum year of birth are less than 5 years, reference year of birth is defined according to the order of data quality of different datasets: APDC > EDDC > RBDM > GPEHR > MHAMB > NAP > CCR > ICD > COD. - If there are recorded dates in general practice data (dates of encounters, chronic disease diagnosis or date of medication prescription) in more than 12 months before year of birth, flag PPN as linkage error and remove PPN. - If there are recorded activities in any dataset in more than 12 months before year of birth, flag PPN as linkage error and remove PPN. - If there are activities in other datasets before the cleansed year of birth but within 1 year window, remove those activities (truncating all activities to year of birth). - If age calculated from derived year of birth is greater than 110, reset age and year of birth as missing. |
| SEIFA Index of Relative Socio-Economic Disadvantage (Deciles) | 1 | APDC/EDDC | - Based on SA1 information. |
|  | 2 | GPEHR | - Based on GPEHR Post Code. |
|  | 3 | Across all datasets | - SA1 based information will be prioritised when constructing SEIFA values. |
| Remoteness | 1 | APDC/EDDC | - Based on SA1 information. |
|  | 2 | GPEHR | - Based on GPEHR Post Code. |
|  | 3 | Across all datasets | - SA1 based information will be prioritised when constructing rurality values. |

## Appendix 6. Demographic variable definition alignments between Lumos and Census 2021

| Variable | Captured | Data dictionary variable definition | Alignment with Census measure |
| --- | --- | --- | --- |
| Sex | Yes | Incomplete | Loss of data due to variation in dataset variables captured and business rules |
| Age | Yes | Complete | Age calculated similarly where date of birth known. Potential minor variation due to differing business rules. |
| IRSD | Yes | Complete | May vary if using GP EHR values as these are derived from postcode. |
| Rurality | Yes | Complete | May vary if using GP EHR values as these are derived from postcode. |

## Appendix 7. Lumos 2022 service utilisation stratified by demographic characteristics

| Total records | General practice attendance N (%) | All hospital admissions N (%) | ED presentations N (%) |
| --- | --- | --- | --- |
| All Lumos | 17,398,698 (100) | 1,257,389 (100) | 1,573,287 (100) |
| Sex |  |  |  |
| *Male* | 7,442,511 (42.8) | 593,522 (47.2) | 760,723 (48.4) |
| *Female* | 9,947,035 (57.2) | 663,680 (52.8) | 812,284 (51.6) |
| Rurality |  |  |  |
| *Major Cities* | 13,546,788 (77.9) | 959,081 (76.3) | 1,052,035 (66.9) |
| *Inner regional* | 2,972,908 (17.1) | 219,310 (17.4) | 355,061 (22.6) |
| *Outer regional* | 734,804 (4.2) | 65,260 (5.2) | 135,845 (8.6) |
| *Remote* | 112,757 (0.6) | 9,733 (0.8) | 25,456 (1.6) |
| IRSD (1 – most relatively disadvantaged areas; 5 – least relatively disadvantaged areas) | | | |
| *1* | 3,436,714 (19.8) | 261,838 (20.8) | 370,002 (24.5) |
| *2* | 3,187,554 (18.3) | 241,265 (19.2) | 347,388 (22.1) |
| *3* | 3,319,245 (19.1) | 246,277 (19.6) | 325,691 (20.7) |
| *4* | 2,516,600 (14.5) | 179,573 (14.3) | 212,164 (13.5) |
| *5* | 4,905,398 (28.2) | 324,339 (25.8) | 312,880 (19.9) |
| Age groups (years) |  |  |  |
| *0-17* | 2,506,830 (14.4) | 112,340 (8.9) | 339,615 (21.6) |
| *18-54* | 6,983,609 (40.1) | 430,936 (34.3) | 723,587 (46.0) |
| *54-74* | 4,677,089 (26.9) | 386,376 (30.7) | 288,121 (18.3) |
| *75 and older* | 3,229,759 (18.6) | 327,737 (26.1) | 221,964 (14.1) |

## Appendix 8. Health service attendance per 100 person-years, 2022

| Total records | General practice  attendance | All hospital admissions | ED presentations |
| --- | --- | --- | --- |
| All Lumos | 530 | 38 | 48 |
| Sex |  |  |  |
| *Male* | 489 | 39 | 50 |
| *Female* | 567 | 38 | 46 |
| Rurality |  |  |  |
| *Major Cities* | 529 | 37 | 41 |
| *Inner regional* | 549 | 40 | 66 |
| *Outer regional* | 494 | 44 | 91 |
| *Remote and very remote* | 522 | 45 | 118 |
| IRSD (1 – most relatively disadvantaged areas; 5 – least relatively disadvantaged areas) | | | |
| *1* | 576 | 44 | 62 |
| *2* | 551 | 42 | 60 |
| *3* | 528 | 39 | 52 |
| *4* | 510 | 36 | 43 |
| *5* | 503 | 33 | 32 |
| Age |  |  |  |
| *0-17* | 383 | 17 | 52 |
| *18-54* | 435 | 27 | 45 |
| *54-74* | 659 | 54 | 41 |
| *75 and older* | 1,043 | 106 | 72 |

## References

1. Weiskopf, N.G., et al., *Defining and measuring completeness of electronic health records for secondary use.* Journal of Biomedical Informatics, 2013. **46**(5): p. 830-836.

2. Declerck, J., et al., *Frameworks, Dimensions, Definitions of Aspects, and Assessment Methods for the Appraisal of Quality of Health Data for Secondary Use: Comprehensive Overview of Reviews.* JMIR Med Inform, 2024. **12**: p. e51560-e51560.

3. Weiskopf, N.G. and C. Weng, *Methods and dimensions of electronic health record data quality assessment: enabling reuse for clinical research.* J Am Med Inform Assoc, 2013. **20**(1): p. 144-151.

4. Terry, A.L., et al., *A basic model for assessing primary health care electronic medical record data quality.* BMC Medical Informatics and Decision Making, 2019. **19**(1): p. 30.

5. Hoeven, L.R.v., et al., *Validation of multisource electronic health record data: an application to blood transfusion data.* BMC Medical Informatics and Decision Making, 2017. **17**(1): p. 107.

6. Kahn, M.G., et al., *A Harmonized Data Quality Assessment Terminology and Framework for the Secondary Use of Electronic Health Record Data.* 2016.
