# Supplementary material for: An assessment of data quality and sociodemographic variation in health service utilisation of general practice, emergency department and admitted services in a New South Wales linked health data asset: a retrospective cohort study of Lumos
Source: BMJ Open. 2025 Jul 22;15(7):e102055. doi: 10.1136/bmjopen-2025-102055 (PMC12306357; doi:10.1136/bmjopen-2025-102055)
Supplement: Supplementary file 2 [file bmjopen-2025-102055.reviewer_comments.pdf]

PEER REVIEW HISTORY

BMJ Open publishes all reviews undertaken for accepted manuscripts. Reviewers are asked to complete a checklist review form and are provided with free text boxes to elaborate on their assessment. These free text comments are reproduced below.

ARTICLE DETAILS

Title (Provisional)

A retrospective cohort study of Lumos, a New South Wales linked health data asset, to assess data quality and sociodemographic variation in health service utilisation of general practice, emergency department and admitted services.

Authors

Bouckley, Tristan; Myton, Rimma; Peiris, David; Nambiar, Devaki; Prince, Samuel; Bishop, Simon; Cordery, Damien; Hill, Flynn Robert; Correll, Pattie; Feyer, Anne-Marie; Schierhout, Gill; Campain, Anna

---

VERSION 1 - REVIEW

**Reviewer**	**1**
**Name**	**Lee, David**
**Affiliation**	**New York University School of Medicine, Emergency Medicine**
**Date**	**09-May-2025**
**COI**	**None**

Overall: This study seeks to evaluate Lumos data on healthcare utilization among Australians. Strengths are that the overall population appears to be well represented in the data nearly 50%. It sounds as if Lumos is an example of a regional information exchange, therefore it might be good to use that sort of verbiage throughout as there is a whole body of literature dedicated to such. Otherwise if not, then explaining why it is different would be helpful.

Abstract: Might be worth explaining what IRSD quintile 4 and 5 are (high or low? and briefly what IRSB represents).

Introduction: Reader does not get a sense of what this area of Australia is like other than it’s the most populous state?

Is Lumos like a regional health information exchange? How is it similar or different? How is healthcare organized in Australia? Is it broken up and federated like the US? Or consolidated and largely government based like other areas of the world?

Some of this context will help the reader understand better how this research study is positioned in the literature.

Methods: How was deduplicated achieved within Lumos if data were coming from different sources? How difficult was the integration of multiple sources of data? Are there issues of missingness or variables that have several different naming conventions? If this is outside of the study methods done (as Lumos is already created). These issues might need to be clarified in the discussion.

Results: Figure 2 legend needs to be much bigger and easy to read.

Discussion: What is the consequence of representativeness of quintiles 4 and 5? Should they be collapsed given these? Also when these are referenced, it is important that the reader can easily understand is this low or high SES?

ED rates are likely higher compared to general Australian population because this is a more urban environment. No distance barrier to utilization.

In rural areas was there any meaningful missingness of general practices? What proportion of practices are included? Is there any bias in this representativeness? (I see this is addressed in some of the important Figures in the Appendix. But these are not discussed so much in the discussion.)

**Reviewer**	**2**
**Name**	**Cornish, Rosie**
**Affiliation**	**University of Bristol, School of Social and Community**
**Medicine**	
**Date**	**12-Jun-2025**
**COI**	**None**

The manuscript is generally clear, but I do not think the title reflects the content – before reading the manuscript I was expecting it to be about patient journeys through the health system, whereas this is not the focus of the manuscript. I would suggest amending the title. I have a few further comments – see below.

Main comments

1. The background section currently suggests (to me) that Lumos is the only database in Australia comprising a linkage between primary care data and state-held health data (e.g. secondary care data). Is this the case? I am aware of large data linkage resources in Western Australia, although admittedly am only aware that these exist and don't know the full details. I appreciate that the focus of this manuscript is to describe the quality/representativeness of the data in Lumos, but think it would be useful to mention other similar resources in different states and how Lumos compares to these.

2. The previous published paper on Lumos (https://doi.org/10.1136/ihj-2021-000074) also reported a comparison of Lumos against the NSW population using census data. I understand that the database has expanded since then, but I think it would be useful to mention this previous comparison, stating whether the findings generally concur (or not, as the case may be).

Minor comments

1. The authors rightly pointed out in the discussion (page 15) that, because Lumos only includes individuals who have attended a (Lumos-enrolled) general practice, it will not necessarily represent the overall population. They state that this would be a "more health conscious and active health seeking population" and also that it would over-represent older people as they generally have higher health service demands. I agree with these statements but also believe that it would over represent individuals with chronic health conditions, females compared to males (particularly in the main childbearing years but also more generally, as indicated by the data), and people who have poor access to services for whatever reason.

VERSION 1 - AUTHOR RESPONSE

**Reviewer 1**	
It sounds as if Lumos is an example of a regional information exchange, therefore it might be good to use that sort of verbiage throughout as there is a whole body of literature dedicated to such. Otherwise if not, then explaining why it is different would be helpful.	The reviewer's comment is correct, the Lumos data asset is a form of health information exchange. However, this terminology is not used so widely to-date in Australia. We have revised to recognise Lumos as a form of HIE, while also retaining the description of Lumos as a linked data asset to ensure consistency within the context of NSW.
Might be worth explaining what IRSD quintile 4 and 5 are (high or low? and briefly what IRSB represents).	Agree, an important element for readers to understand. We have included a high-level definition in both the abstract and methods.
Reader does not get a sense of what this area of Australia is like other than it's the most populous state	We have revised to include further relevant information in the fourth paragraph in the background, including details on chronic health conditions and the broader health context of NSW.
How is healthcare organized in Australia? Is it broken up and federated like the US? Or consolidated and largely government based like other areas of the world?	We have included further details on the Australian health system in the second paragraph in the background. Recognising that it has a relatively unique mixed model design.

How was deduplicated achieved within Lumos if data were coming from different sources?	We have tweaked the first paragraph of the data consistency section to clarify duplicated input data which may differ between sources. We have detailed how this is overcome in appendix 5.
How difficult was the integration of multiple sources of data?	Integration of data sources was conducted by the Ministry of Health, and the process previously reported which we refer to in the data sources section.
Are there issues of missingness or variables that have several different naming conventions?	Where important difference in naming conventions have occurred, we have highlighted the risk in the discussion as this is a significant problem for sex. For others, naming variations is not an issue, as either the variables are captured the same or we have clear rules detailed in appendix 5 to clarify how each variable is determined and which data set is prioritised should conflict occur. We have reported any missing data for each variable int able 1.
Figure 2 legend needs to be much bigger and easy to read.	We agree with the reviewer and have updated to increase size. We have also revised the legend in appendix 4 to align.
Discussion: What is the consequence of representativeness of quintiles 4 and 5? Should they be collapsed given these? Also when these are referenced, it is important that the reader can easily understand is this low or high SES?	We have revised to clarify what the IRSD quintiles represent in paragraph 3 of the data quality section within the Methods as well as defined the index of SES within the discussion to support readers. We do not believe these should be collapsed, as the quintiles represent meaningful difference in population SES. We reflect in the discussion that this can be overcome where required.
ED rates are likely higher compared to general Australian population because this is a more urban environment. No distance barrier to utilization.	We have revised to clarify that this should be considered in comparison to other New South Wales reported data. We don't think this is necessarily driven by a more urban environment, as in Australia, ED utilisation is often reported to be greater in less urban areas. However, as we note, this is often correlated with greater socioeconomic disadvantage.
In rural areas was there any meaningful missingness of general practices? What proportion of practices are included? Is there any bias in this representativeness? (I see this is addressed in some of the important Figures in	We agree with the reviewer, there are considerations that need to be recognised in the missingness of GPs and bias. As the reviewer raises, we have

the Appendix. But these are not discussed so much in the discussion.)	highlighted this in Appendix 4. We further discuss this challenge within the fourth paragraph of the discussion
**Reviewer 2**	
I do not think the title reflects the content – before reading the manuscript I was expecting it to be about patient journeys through the health system, whereas this is not the focus of the manuscript. I would suggest amending the title.	In line with the journal requirements and the reviewer's comment, we have revised our title to be more specific towards the methods and aims.
The background section currently suggests (to me) that Lumos is the only database in Australia comprising a linkage between primary care data and state-held health data (e.g. secondary care data). Is this the case? I am aware of large data linkage resources in Western Australia, although admittedly am only aware that these exist and don't know the full details. I appreciate that the focus of this manuscript is to describe the quality/representativeness of the data in Lumos, but think it would be useful to mention other similar resources in different states and how Lumos compares to these.	While there has been considerable data linkage previously of state held hospital dataset, population-wide data linkage between primary care and state held data is still relatively new to Australia. That said, at the time of writing there is now availability of access at a national level of large, linked dataset (National health data hub). We have revised the paper to reflect this and a few other relevant data linkages that are most relevant.
The previous published paper on Lumos (https://doi.org/10.1136/ihj-2021-000074) also reported a comparison of Lumos against the NSW population using census data. I understand that the database has expanded since then, but I think it would be useful to mention this previous comparison, stating whether the findings generally concur (or not, as the case may be).	This is a good point to raise, and we agree that it would be beneficial to the reader. We have revised the discussion to include a section recognising alignments with the early study.
The authors rightly pointed out in the discussion (page 15) that, because Lumos only includes individuals who have attended a (Lumos-enrolled) general practice, it will not necessarily represent the overall population. They state that this would be a "more health conscious and active health seeking population" and also that it would over-represent older people as they generally have higher health service demands. I agree with these statements but also believe that it would over represent individuals with chronic health conditions, females compared to males (particularly in the main childbearing years but also more generally, as indicated by the data), and people who have poor access to services for whatever reason.	We agree with the reviewers' considerations here and have expanded to incorporate the suggestions. This is particularly relevant to females of childbearing age, which aligns with the findings in our age and sex pyramid.
